# Preventive treatment can reverse cognitive impairment in chronic migraine

**DOI:** 10.1186/s10194-022-01486-w

**Published:** 2022-09-15

**Authors:** Cristina González-Mingot, Anna Gil-Sánchez, Marc Canudes-Solans, Silvia Peralta-Moncusi, Maria José Solana-Moga, Luis Brieva-Ruiz

**Affiliations:** 1 Hospital Universitari Arnau de Vilanova, Lleida, Spain; 2 Biomedical Research Institute of Lleida, Lleida, Spain

## Abstract

**Objective:**

To study the impact of chronic migraine (CM) on the cognition and quality of life (QoL) of patients in the interictal period, and to analyse the degree of reversibility of any observed alterations following the use of preventive treatment.

**Background:**

CM is a highly disabling disease, and migraineurs often have associated comorbidities, such as subjective memory problems, that are involved in the development of cognitive impairment. Our hypotheses are that patients suffering from chronic migraine experience objective cognitive alterations that are not only due to the pain that they suffer or their current emotional state. Furthermore, preventive treatment should be capable of reversing, or at least reducing, the impact of CM on the cognition and QoL of migraineurs.

**Methods:**

The cognition and QoL of 50 control subjects and 46 patients with CM were assessed using a battery of tests, prior to the use of preventive treatment based on botulinum toxin or oral drugs and after 3 months of this treatment.

**Results:**

Compared with controls, patients with CM had lower scores on the assessment of cognitive performance (Rey-Osterrieth Complex Figure test [ROCF] *(p<0.05)*, Trail Making Test [TMT] B) (*p* < 0.05) and QoL (*p* < 0.05). Three months after the use of preventive treatment, improvement was observed in all cognitive parameters (*p* < 0.05) and QoL (*p* < 0.05), except the ROCF copy task (*p* = 0.79). No statistically significant differences were observed when these outcomes were compared based on treatment.

**Conclusions:**

This study confirms poor cognitive performance that is not explained by migraine pain itself, as it occurs in the interictal period, irrespective of the patient’s emotional status. Our findings show that these effects are reversible in some cases with preventive treatment of CM, reaffirming the important impact of this condition on the QoL of these patients, and the need to establish preventive treatment guidelines.

**Supplementary Information:**

The online version contains supplementary material available at 10.1186/s10194-022-01486-w.

## Introduction

Chronic migraine (CM) is a complication of migraine that is defined by the presence of ≥ 15 headache days per month for at least 3 months, with at least 8 of those days meeting criteria for migraine, in the absence of medication overuse and other causes to which the headache could be attributed [1].

The estimated global prevalence of migraine is 14.0% [2]. It is the second leading cause of disability worldwide, and the first among young women [3]. It usually begins episodically, with the nervous system returning to a normal or premorbid functional state between attacks. However, approximately 2.5% of patients with episodic migraine experience transformation to CM [4]. The pathophysiology of CM and the mechanisms responsible for migraine chronification are not fully understood, although atypical pain processing during migraine episodes, central sensitization, cortical hyperexcitability, and neurogenic inflammation have been proposed as possible causes [3].

Management of CM consists of acute treatment of attacks with non-steroidal anti-inflammatory drugs or triptans, and preventive treatment to try to reduce the frequency, duration and intensity of attacks. Topiramate, beta-blockers, amitriptyline, and flunarizine are oral drugs commonly used as preventive agents in CM [5]. Within the injectable drug group, the use of botulinum toxin has been found to be beneficial in 75% of cases, and positive results have also been reported for anti-calcitonin gene-related peptide monoclonal antibodies in refractory cases [6, 7].

Patients with CM have more intense headaches and more severe symptoms than patients with episodic migraine, and the impact on their quality of life (QoL) is greater [8]. Some epidemiological studies have suggested that migraine is bidirectionally associated with several other disorders, including psychiatric disorders and chronic fatigue. It has also been associated with other pain-causing conditions, such as fibromyalgia, that are responsible for non-painful disabilities associated with migraine [9, 10]. Certain functional neurologic disorders, like psychogenic non-epileptic episodes and other functional movement-related disorders have also been linked to migraine and attempted suicides have also been reported amongst relatively young migraine patients [10, 11].

Migraine patients also frequently attend consultations for subjective cognitive complaints that are mainly attention- and memory-related and appear during or after migraine attacks, and contribute to the disability of patients experiencing them [12, 13]. Some factors related to CM, such as preventive therapies and comorbidities like depression, anxiety, and sleep disturbance, may contribute to the development of these cognitive deficits, although they are not the only cause of cognitive impairment in migraineurs [14, 15]. It has been suggested that certain biological mechanisms may be shared between migraine, fatigue, mood and cognition. Rather than true comorbidities, these mechanisms may be the reason for these associations. It is, however, currently unknown whether the treatment of migraine could influence the associated alterations [9–11]. Although several clinically based studies of patients with CM have described interictal impairment of cognitive function [16–18], results from studies based on other populations differ [19–21]. Longitudinal studies do not suggest any progressive decline in cognitive function [22, 23], while existing studies analysing cognitive performance in patients with CM have been generally limited in scope and number [15, 16, 24].

The functional organization of the neural networks associated with cognitive and pain processes may be altered by migraine at different levels, including pain processing and modulation. The impairment of structural network integrity increases with the presence of migraine attacks, indicating that CM causes the strongest structural abnormalities [25]. Affective, emotional and cognitive processing is also affected, specifically in the hippocampus, parahippocampal gyrus and orbitofrontal cortex, and other areas in which differences have been identified on functional magnetic resonance imaging (MRI) in CM patients [26–30]. These structural changes are already seen in patients with a migraine frequency of > 9 days per month [31]. Other cognitive domains affected in these patients include processing speed, attention span, memory, verbal skills, and executive function processes (working memory, divided attention, and planning) [32].

Despite evidence of the cognitive deficit associated with CM, few studies have been conducted in the context of cognitive impairments between attacks or on the effect of preventive treatment of CM on the reversibility of these impairments, emotional status and QoL. It is also still unclear whether the comorbidities present in patients with CM contribute indirectly to cognitive dysfunction or whether the underlying pathophysiological process of CM directly affects cognition. Given the high prevalence of migraine in the population, establishing an association between CM and cognitive decline could have substantial public health implications. Our hypotheses are that patients suffering from chronic migraine experience objective cognitive alterations that are not only due to the pain that they suffer or their current emotional state. Furthermore, preventive treatment should be capable of reversing, or at least reducing, the impact of CM on the cognition and QoL of migraineurs. With this in mind, the present study analyses impaired cognition in CM and the impact of this impairment on the QoL of CM patients before and after receiving preventive treatment based on oral therapies or botulinum toxin.

## Methods

We conducted an observational study in the headache clinic at Hospital Universitari Arnau de Vilanova (Lleida, Spain) to analyse factors related to the cognitive status and QoL of patients with CM before receiving preventive treatment with botulinum toxin or oral drugs commonly used in the management of CM, and 3 months after receiving any of these therapies.

### Study population

The study was approved by the ethics committee of Hospital Universitari Arnau de Vilanova (CEIC-2073), and informed consent was obtained from all participants. The cognitive status and QoL of 46 patients with CM and 50 healthy controls were evaluated. There were no significant differences in the age, sex or level of education of the subjects included in the groups. The CM patient group included individuals with normal brain MRI scans and CM diagnosed according to International Classification for Headache Disorders-III criteria [33]. Preventive treatment was initiated in these patients (treatment-naïve) or a second preventive drug was added if > 50% efficacy was not achieved with previous therapies. Patients with CM who had an oncological, inflammatory, or neurodegenerative disease and patients with decompensated psychiatric disease or who had experienced changes in the past 3 months if they were receiving psychiatric treatment were excluded (Supplementary Table S1).

### Variables analysed

The factors analysed in this study were assessed in the interictal period so that acute pain did not interfere with the study results. We analysed general (age, sex, education level and work activity) and clinical variables (intensity and frequency of attacks pre- and post-treatment, age at onset of migraine, preventive treatment, acute treatment of attacks).

Variables related to mood, QoL, and cognition were also analysed. For mood, we used the Hospital Anxiety and Depression Scale [HADS] [34], which is one of the scales most commonly used in trials to evaluate the combination of anxiety and depression, and also the Beck Depression Inventory-II [BDI-II] [35], which combines 21 items that measure the degree to which people feel sorry or guilty, lack energy, and note changes in their ability to do tasks in their real life. For QoL we used the 36-item Short Form Health Survey [SF36], which includes 36 items that are often used in trials, and also the Pittsburgh Sleep Quality Index [PSQI] [36, 37], to assess healthy sleep. For cognition, we used the Rey-Osterrieth Complex Figure test [ROCF] copy and reproduction task. We were therefore able to measure executive functions and visuospatial ability. With the second part of the test, we measured memory (ROCF memory task [ROCF-m] [38, 39] and used the Taylor Complex Figure Test for longitudinal evaluation [40] to avoid the learning effect. We then used the Trail Making Test (TMT) A and B [41] to measure attention and information processing speed (IPS). These tasks consist of finding ordered numbers on a sheet of paper and then, for the second part of the test, relating ordered numbers to letters of the alphabet which are also in order. This gave us a measure of working memory with which to make our evaluation.

A description of the sample and comparison with the controls was performed to evaluate the presence of cognitive impairments in the interictal period. Baseline (pre-treatment) and longitudinal (post-treatment) analyses were conducted to (i) analyse whether the cognitive impairment in CM are reversible after preventive treatment, (ii) determine the impact of these impairments on the QoL of CM patients, and (iii) study the differences in cognition and QoL of patients according to the drugs used for the treatment of CM.

To this end, descriptive analyses and comparison of the differences between the CM and control groups (Mann–Whitney U test) were performed at baseline. We used the Wilcoxon test to determine the changes resulting from the treatment at the longitudinal level.

## Results

### Sample description

The study included 46 CM patients, 40 of whom completed the study, and 50 controls of similar sex and age. The mean age of the sample was 47.17 years in the CM group and 47.82 years in the control group. No significant differences were found in the educational profile of the participants or in the duration of studies of the individuals included in each group (12.41 ± 4.76 years in the CM group and 13.72 ± 3.07 years in the control group). The measurement with respect to the time since onset of CM in the patients was 3.42 ± 4.34 years. Two-thirds of patients (67%) with CM were treatment-naïve, while the remaining 33% were already receiving oral preventive treatment at the time of inclusion in the study.

With regard to the preventive treatments used, 28.6% of patients used oral treatment only (beta-blockers, 50 mg/day; topiramate, 25 mg/12 h), 41.3% of cases used botulinum toxin only, and in 30.43% of cases, botulinum toxin was added to previous oral treatment (Table 1).

**Table 1:** Preventive treatments scheme (*n* = 46; 67% patients, treatment-naïve)

Preventive treatment	*n* (%)
Botulinum toxin	**19 (41.3)**
Oral treatments	**13 (28.26)**
BB	3 (6.52)
TPM	10 (21.74)
Botulinum toxin + oral treatments	**14 (30.43)**
Botulinum toxin + BB	3 (6.52)
Botulinum toxin + TPM	4 (8.7)
Botulinum toxin + FNZ	4 (8.7)
Botulinum toxin + BB + TPM	3 (6.52)

*BB* beta-blockers, *FNZ* flunarizine, *TPM* topiramate

^a^BB, 50 mg/day; TPM, 25 mg/12 h; or FNZ, 10 mg/day

Regarding the response to preventive therapy after 3 months of treatment, a > 50% improvement was found in terms of a reduction in both the number of days and intensity of migraine in 79% of cases (*p* < 0.0001). No differences were found in the response rate in the treatment groups; the > 50% reduction in number of migraine days occurred in 85% of CM patients in whom oral preventive treatment was initiated, and in 83% of cases in which botulinum toxin was used.

### Comparison between patients with CM and control subjects at baseline

**Fig. 1:**
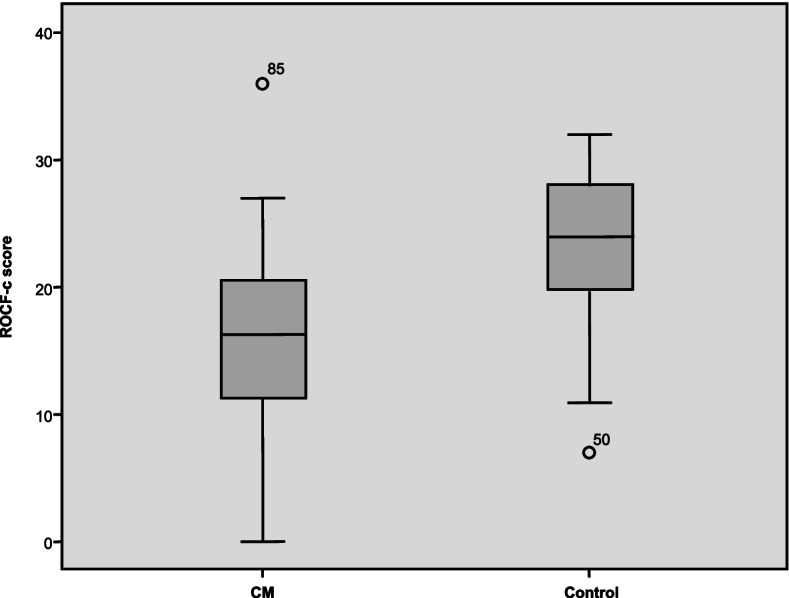
Results of the ROCF copy task in the CM group and the control group. ROCF-c: Rey-Osterrieth Complex Figure test, copy task; CM: chronic migraine

**Fig. 2:**
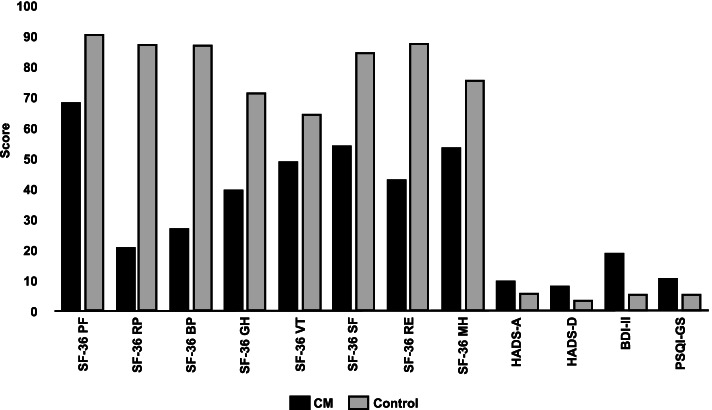
Results of the QoL assessment in the CM group and the control group. Results were evaluated for the SF-36, HADS, PSQI, and BDI scales. QoL: quality of life; CM: chronic migraine; SF-36 PF: 36-item Short Form Health Survey, physical functioning; SF-36 RP: role-physical; SF-36 BP: bodily pain; SF-36 GH: general health; SF-36 VT: vitality; SF-36 SF: social functioning; SF-36 RE: role-emotional; SF-36 MH: mental health; BDI-II: Beck Depression Inventory II; PSQI-GS: Pittsburgh Sleep Quality Index, global score

Analysis of the differences between patients with CM and individuals in the control group was statistically significant for all the cognitive parameters analysed, QoL, and emotional status, with worse scores in the CM group (*p* < 0.0001) (Table 2, Figs. 1 and 2). No differences were observed between patients according to the drug used (*p* = 0.79).

**Table 2:** Comparison between the CM group (*n* = 46) and the control group (*n* = 50) in the cognition and quality of life scales used at baseline

	**CM** **(*n* = 46)** $\overline{\mathbf{x}}$** (SD)**	**Control group** **(*n* = 50)** $\overline{\mathbf{x}}$** (SD)**	***P* Value**
**Cognitive measures**			
ROCF-c	30.09 (5.4)	33.1 (2.84)	0.001
ROCF-m	15.78 (7.01)	23.22 (5.9)	0.000
TMT-A	56.79 (48.25)	43.7 (38.72)	0.016
TMT-B	112.04 (49.99)	90.84 (72.03)	0.004
**Emotional state**			
HADS—A	9.69 (4.04)	5.35 (2.83)	0.000
HADS—D	7.91 (4.71)	2.78 (2.54)	0.000
BDI-II	18.2 (13.4)	5.2 (4.88)	0.000
**Quality of life**			
SF-36 PF	67.93 (22.42)	90.5 (19.15)	0.000
SF-36 RP	20.65 (31.76)	87.2 (28.98)	0.000
SF-36 BP	26.58 (23.16)	86.75 (91.48)	0.000
SF-36 GH	39.24 (19.63)	71.2 (16.65)	0.000
SF-36 VT	48.57 (81.38)	64.27 (19.41)	0.000
SF-36 SF	53.53 (26.44)	84.5 (20.6)	0.000
SF- 36 RE	42.78 (46.98)	87.34 (26.87)	0.000
SF-36 MH	53.11 (19.61)	75.32 (16)	0.000
PSQI-GS	10.49 (4.65)	5.28 (3.47)	0.000

*ROCF-c* Rey-Osterrieth Complex Figure test, copy task, *ROCF-m* ROCF, memory task, *TMT-A* Trail Making Test A, attention test, *TMT-B* Trail Making Test B, divided attention test, *HADS-A* Hospital Anxiety and Depression Scale, anxiety subscale, *HADS-D* Hospital Anxiety and Depression Scale, depression subscale, *BDI-II* Beck Depression Inventory II, *SF-36 PF* 36-item Short Form Health Survey, physical functioning, *SF-36 RP* role-physical, *SF-36 BP* bodily pain, *SF-36 GH* general health, *SF-36 VT* vitality, *SF-36 SF* social functioning, *SF-36 RE* role-emotional, *SF-36 MH* mental health, *PSQI-GS* Pittsburgh Sleep Quality Index, global score

### Comparison between baseline and 3 months after treatment with preventive therapies in patients with CM

**Fig. 3:**
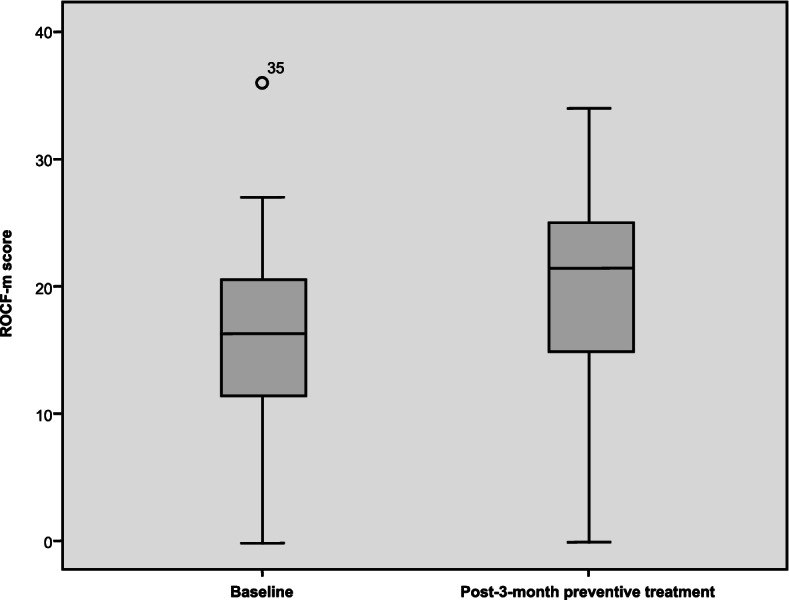
Results of the ROCF memory task in the CM group at baseline and 3 months after preventive treatment. ROCF-m: Rey-Osterrieth Complex Figure test, memory task

The differences in the results of the ROCF-m and TMT A tests after 3 months of treatment were statistically significant (*p* < 0.0001; *p* = 0.007), as were those comparing QoL (SF-36 bodily pain, *p* = 0.040; and SF-36 general health, *p* = 0.003); and PSQI, *p* = 0.025) before and after treatment (Table 3, Fig. 3). There was also a trend toward an improvement in depression (*p* = 0.052), although it did not reach statistical significance.

**Table 3:** Quality of life and cognition test scores before and three months after the start of preventive treatment in patients with CM

	**Pre-treatment** **(*n* = 46)** $\overline{\mathbf x}\;(\mathbf S\mathbf D)$	**Post-treatment** **(*n* = 40)** $\overline{\mathbf x}\;(\mathbf S\mathbf D)$	***P* Value**
**Migraine**			
Frequency	21.58 (5.96)	9.54 (9.8)	0.000
Intensity	8.25 (1.42)	6.24 (1.98)	0.000
**Cognitive measures**			
ROCF-c / TFT-c	30.09 (5.4)	30.98 (4.95)	0.227
ROCF-m / TFT-m	15.78 (7.01)	19.6 (7.55)	0.000
TMT-A	56.79 (48.25)	44.14 (30.31)	0.007
TMT-B	112.04 (49.99)	104.19 (56.88)	0.080
**Emotional state**			
HADS—A	9.69 (4.04)	9.38 (4.79)	0.485
HADS—D	7.91 (4.71)	7.45 (4.86)	0.171
BDI-II	18.2 (13.4)	15.23 (13.52)	0.052
**Quality of life**			
SF-36 PF	67.93 (22.42)	69.73 (25.74)	0.679
SF-36 RP	20.65 (31.76)	28.75 (36.93)	0.305
SF-36 BP	26.58 (23.16)	31.48 (19.07)	0.040
SF-36 GH	39.24 (19.63)	45.13 (19.82)	0.003
SF-36 VT	48.57 (81.38)	39.85 (18)	0.830
SF-36 SF	53.53 (26.44)	58.75 (26.88)	0.386
SF- 36 RE	42.78 (46.98)	60 (43.51)	0.091
SF-36 MH	53.11 (19.61)	57.33 (21.22)	0.294
PSQI-GS	10.49 (4.65)	8.97 (4.82)	0.025

*ROCF-c* Rey-Osterrieth Complex Figure test, copy task, *ROCF-m* Rey-Osterrieth Complex Figure test, memory task, *TFT-c* Taylor Figure test, copy task, *TFT-m* Taylor Figure test, memory task, *TMT-A* Trail Making Test A, attention test, *TMT-B* Trail Making Test B, divided attention test, *HADS-A* Hospital Anxiety and Depression Scale, anxiety subscale, *HADS-D* Hospital Anxiety and Depression Scale, depression subscale, *BDI-II* Beck Depression Inventory II, *SF-36 PF* 36-item Short Form Health Survey, physical functioning, *SF-36 RP* role-physical, *SF-36 BP* bodily pain, *SF-36 GH* general health, *SF-36 VT* vitality, *SF-36 SF* social functioning, *SF-36 RE* role-emotional, *SF-36 MH* mental health, *PSQI-GS* Pittsburgh Sleep Quality Index, global score

### Comparison between patients with CM and control subjects after 3 months of treatment with preventive therapies in CM patients

**Fig. 4:**
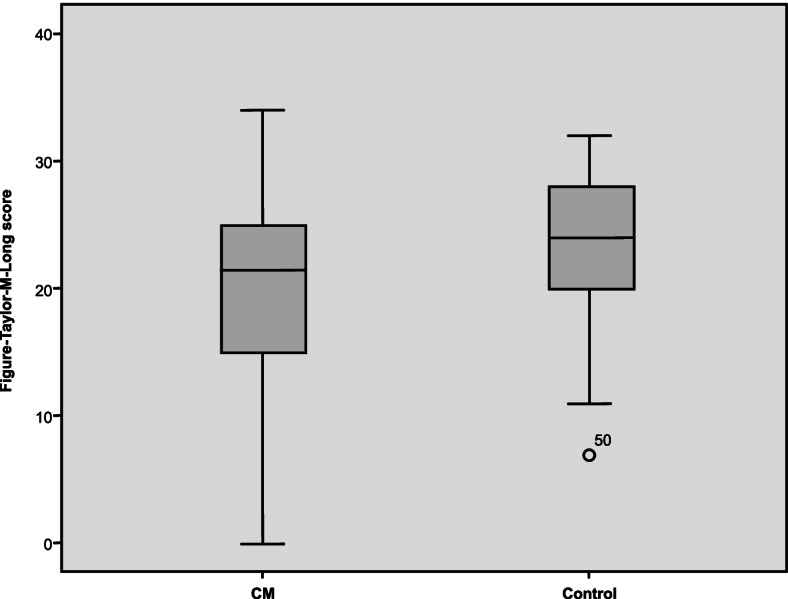
Results of the Taylor memory task in the CM group and the control group after 3 months of treatment with preventive therapies. Figure-Taylor-M-Long: Taylor Figure test, memory task (longitudinal); CM, chronic migraine

To determine if CM patients who improved after receiving preventive therapies could achieve the level of cognitive performance of the controls, we examined cognition and QoL parameters in CM patients after 3 months of preventive treatment compared with controls. We found that after 3 months of preventive treatment, CM patients only reached normal values in the TMT A (*p* = 0.561), whereas the differences between the patients and the controls in the rest of the study variables remained statistically significant (Table 4, Fig. 4).

**Table 4:** Comparison between the CM group after three months of preventive treatment (*n* = 40) and the control group (*n* = 50) in the cognition and quality of life scales used

	**CM Post-treatment** **(*n* = 40)** $\overline{\mathbf x}\;(\mathbf S\mathbf D)$	**Control group** **(*n* = 50)** $\overline{\mathbf x}\;(\mathbf S\mathbf D)$	***P* Value**
**Cognitive measures**			
TFT-c	30.98 (4.95)	33.1 (2.84)	0.001
TFT-m	19.6 (7.55)	23.22 (5.9)	0.022
TMT-A	44.14 (30.31)	43.7 (38.72)	0.561
TMT-B	104.19 (56.88)	90.84 (72.03)	0.157
**Emotional state**			
HADS—A	9.38 (4.79)	5.35 (2.83)	0.000
HADS—D	7.45 (4.86)	2.78 (2.54)	0.000
BDI-II	15.23 (13.52)	5.2 (4.88)	0.000
**Quality of life**			
SF-36 PF	69.73 (25.74)	90.5 (19.15)	0.000
SF-36 RP	28.75 (36.93)	87.2 (28.,98)	0.000
SF-36 BP	31.48 (19.07)	86.75 (91.48)	0.000
SF-36 GH	45.13 (19.82)	71.2 (16.65)	0.000
SF-36 VT	39.85 (18)	64.27 (19.41)	0.000
SF-36 SF	58.75 (26.88)	84.5 (20.6)	0.000
SF- 36 RE	60 (43.51)	87.34 (26.87)	0.002
SF-36 MH	57.33 (21.22)	75.32 (16)	0.000
PSQI-GS	8.97 (4.82)	5.28 (3.47)	0.000

*TFT-c* Taylor Figure test, copy task, *TFT-m* Taylor Figure test, memory task, *TMT-A* Trail Making Test A, attention test, *TMT-B* Trail Making Test B, divided attention test, *HADS-A* Hospital Anxiety and Depression Scale, anxiety subscale, *HADS-D* Hospital Anxiety and Depression Scale, depression subscale, *BDI-II* Beck Depression Inventory II, *SF-36 PF* 36-item Short Form Health Survey, physical functioning; SF-36 RP: role-physical; SF-36 BP: bodily pain; *SF-36 GH* general health; *SF-36 VT* vitality, *SF-36 RP* social functioning, *SF-36 RE* role-emotional, *SF-36 MH* mental health, *PSQI-GS* Pittsburgh Sleep Quality Index, global score

Furthermore, a group of 15 patients with cognitive impairment showed no significant improvement despite effective preventive treatment. We found that this lack of cognitive improvement was due not to a higher frequency or intensity of migraine, but to the greater presence of anxiety (*p* = 0.03) and depression (*p* = 0.02), which was associated with a lower level of education (*p* = 0.019).

### Differences in cognitive performance between patients with CM and controls without mood alterations at baseline

**Fig. 5:**
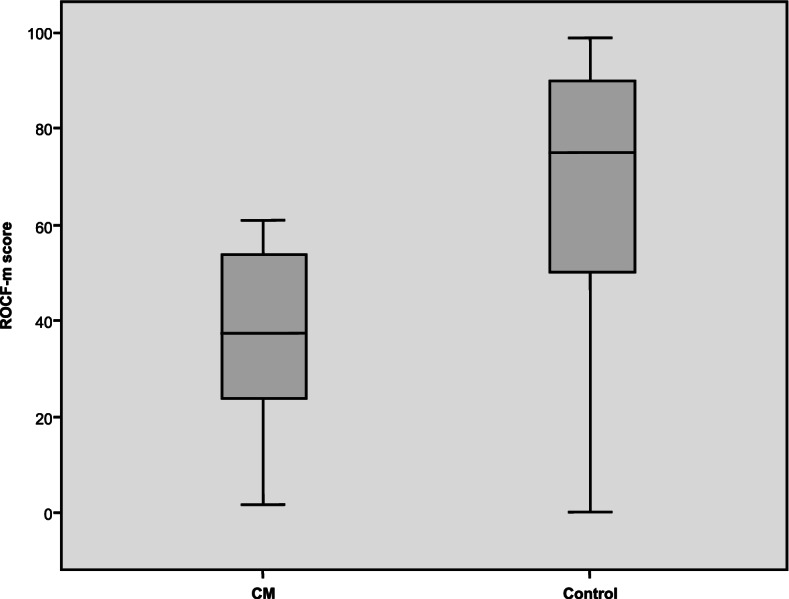
Results of the ROCF memory task in the CM group and the control group without mood changes at baseline. ROCF-m: Rey-Osterrieth Complex Figure test, memory task

We examined the impact of mood alterations on cognitive performance in CM patients. To this end, the sample of CM patients and controls was divided into those with normal mood, defined as scores < 9 on HADS and < 10 on BDI (*n* = 14), and cases with altered mood with higher scores (*n* = 32). The comparison between CM patients and controls without mood alterations revealed statistically significant differences in the ROCF-m test (*p* = 0.001), SF-36 score (*p* = 0.001), and in the variables analysed for depression (*p* = 0.004) and sleep (*p* < 0.05) (Table 5, Fig. 5).

**Table 5:** Comparison between patients with normal mood in the CM group (*n* = 14) and the control group (*n* = 50) at baseline

	**CM NMA** **(*n* = 14)** $\overline{\mathbf x}\;(\mathbf S\mathbf D)$	**Control group** **(*n* = 50)** $\overline{\mathbf x}\;(\mathbf S\mathbf D)$	***P* Value**
**Cognitive measures**			
ROCF-c	3261 (3.1)	33.1 (2.96)	0.504
ROCF-m	18.46 (4.01)	23.49 (5.45)	0.001
TMT-A	45.89 (20.89)	42.61 (39.62)	0.104
TMT-B	92.97 (44.85)	82.37 (58.95)	0.111
**Emotional state**			
HADS—A	5.64 (2.53)	4.77 (2.42)	0.165
HADS—D	3.5 (1.7)	2.02 (1.77)	0.004
BDI-II	5.64 (1.6)	3.71 (3.3)	0.040
**Quality of life**			
SF-36 PF	80 (14.14)	90.85 (19.39)	0.000
SF-36 RP	35.71 (38.87)	86.22 (31.5)	0.000
SF-36 BP	35.36 (18.93)	92.62 (99.79)	0.000
SF-36 GH	55.36 (12.16)	73.05 (17.32)	0.001
SF-36 VT	48.04 (17.02)	67.34 (17.57)	0.001
SF-36 SF	74.11 (24.25)	89.02 (16.34)	0.023
SF- 36 RE	59.55 (47.47)	91.07 (22.4)	0.006
SF-36 MH	66 (19.3)	78.46 (12.41)	0.022
PSQI-GS	7.08 (3.68)	4.98 (3.31)	0.026

*NMA* No mood alterations *ROCF-c* Rey-Osterrieth Complex Figure test, copy task, *ROCF-m* ROCF, memory task, *TMT-A* Trail Making Test A, attention test, *TMT-B* Trail Making Test B, divided attention test, *HADS-A* Hospital Anxiety and Depression Scale, anxiety subscale, *HADS-D* Hospital Anxiety and Depression Scale, depression subscale, *BDI-II* Beck Depression Inventory II, *SF-36 PF* 36-item Short Form Health Survey, physical functioning, *SF-36 RP* role-physical, *SF-36 BP* bodily pain, *SF-36 GH* general health, *SF-36 VT* vitality, *SF-36 SF* social functioning, *SF-36 RE* role-emotional, *SF-36 MH* mental health, *PSQI-GS* Pittsburgh Sleep Quality Index, global score

### Differences in cognitive performance based on patient mood before and after 3 months of preventive treatment

**Fig. 6:**
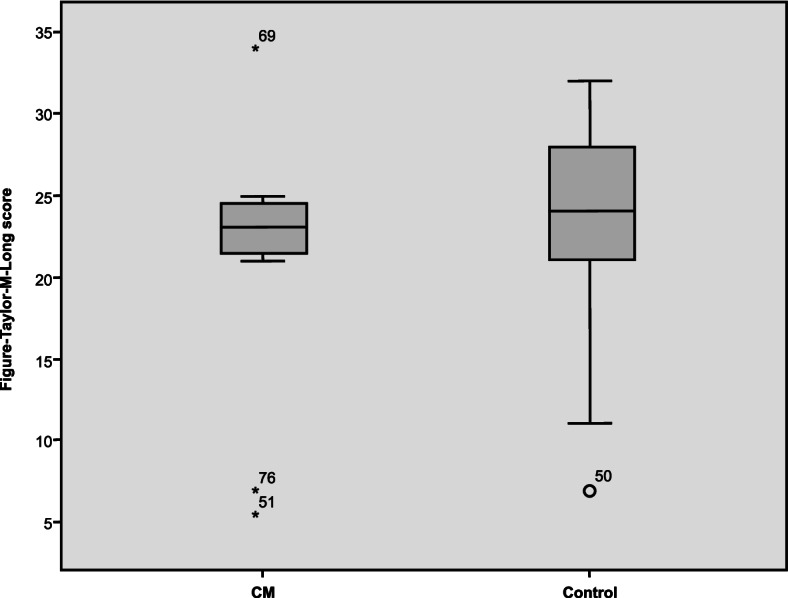
Results of the assessment of the Taylor memory task in the subgroups of patients with CM and normal mood 3 months after preventive treatment and of control subjects with normal mood. Figure-Taylor-M-Long: Taylor Figure test, memory task (longitudinal); CM, chronic migraine

When comparing the pre- and post-preventive treatment results in the evaluation of cognition and QoL in the subgroup of patients with CM and normal mood, an improvement was observed in all the parameters analysed, although these differences were not statistically significant. In this case, we found that patients who showed improvement achieved the scores of the controls in the Taylor Figure Test for longitudinal evaluation (Table 6, Fig. 6).

**Table 6:** Results of the quality of life and cognition tests in the subgroup of patients with CM and normal mood at baseline (*n* = 14) and after three months of preventive treatment (*n* = 12)

	**CM** **Pre-treatment** **(*n* = 14)** $\overline{\mathbf x}\;(\mathbf S\mathbf D)$	**CM** **Post-treatment** **(*n* = 12)** $\overline{\mathbf x}\;(\mathbf S\mathbf D)$	***P* Value**
**Migraine**			
Frequency	22.57 (5.49)	12.75 (10.75)	0.018
Intensity	7.43 (1.24)	5.95 (1.88)	0.053
**Cognitive measures**			
ROCF-c / TFT-c	32.61 (3.1)	33.25 (2.09)	0.888
ROCF-m / TFT-m	18.46 (4.01)	21.38 (7.78)	0.075
TMT-A	45.89 (20.89)	49.33 (46.04)	0.068
TMT-B	92.97 (44.85)	104.25 (99.18)	0.530
**Emotional state**			
HADS—A	5.64 (2.53)	5.75 (3.28)	0.730
HADS—D	3.5 (1.7)	3.42 (2.78)	0.187
BDI-II	5.64 (1.6)	5.42 (4.93)	0.422
**Quality of life**			
SF-36 PF	80 (14.14)	87.5 (12.52)	0.190
SF-36 RP	35.71 (38.87)	54.17 (41.06)	0.194
SF-36 BP	35.36 (18.93)	44.92 (15.2)	0.195
SF-36 GH	55.36 (12.16)	58.33 (14.97)	0.279
SF-36 VT	48.04 (17.02)	53.54 (17.44)	0.196
SF-36 SF	74.11 (24.25)	83.33 (17.94)	0.427
SF- 36 RE	59.55 (47.47)	77.75 (35.81)	0.465
SF-36 MH	66 (19.3)	75.58 (12.28)	0.060
PSQI-GS	7.08 (3.68)	6.5 (3.97)	0.403

*ROCF-c* Rey-Osterrieth Complex Figure test, copy task, *ROCF-m* Rey-Osterrieth Complex Figure test, memory task, *TFT-c* Taylor Figure test, copy task, *TFT-m* Taylor Figure test, memory task, *TMT-A* Trail Making Test A, attention test, *TMT-B* Trail Making Test B, divided attention test, *HADS-A* Hospital Anxiety and Depression Scale, anxiety subscale, *HADS-D* Hospital Anxiety and Depression Scale, depression subscale, *BDI-II* Beck Depression Inventory II, *SF-36 PF* 36-item Short Form Health Survey, physical functioning, *SF-36 RP* role-physical, *SF-36 BP* bodily pain, *SF-36 GH* general health, *SF-36 VT* vitality, *SF-36 SF* social functioning, *SF-36 RE* role-emotional, *SF-36 MH* mental health, *PSQI-GS* Pittsburgh Sleep Quality Index, global score

### Comparison between patients with CM according to time since onset of migraine

Patients with CM were divided into 2 groups according to the time since onset of migraine (0–2 years, *n* = 23; > 2 years, *n* = 17). Although no statistically significant differences were observed in any of the variables analysed, patients with > 2 years since onset obtained better scores than those with migraine 0–2 years since onset (Table 7).

**Table 7:** Comparison of the parameters analysed in relation to cognition and quality of life in patients with CM with 0–2 years since onset of migraine (*n* = 23) or patients with CM with > 2 years since onset (*n* = 17)

	**CM 0–2 years** **(*n* = 23)** $\overline{\mathbf x}\;(\mathbf S\mathbf D)$	**CM > 2 years** **(*n* = 17)** $\overline{\mathbf x}\;(\mathbf S\mathbf D)$	***P* Value**
**Migraine**			
Frequency	21.73 (6.07)	21.37 (5.96)	0.844
Intensity	8.13 (1.57)	8.4 (1.23)	0.667
**Cognitive measures**			
ROCF-c	29.33 (5.48)	31.08 (5.26)	0.217
ROCF-m	14.94 (7.81)	16.88 (5.82)	0.202
TMT-A	63.58 (57.34)	47.97 (32.28)	0.187
TMT-B	120.88 (48.69)	100.55 (50.53)	0.067
**Emotional state**			
HADS—A	10.27 (4.41)	8.89 (3.45)	0.337
HADS—D	8.12 (5.31)	7.63 (3.86)	0.945
BDI-II	18.5 (14.91)	17.79 (11.39)	0.730
**Quality of life**			
SF-36 PF	62.5 (23.16)	75 (19.8)	0.081
SF-36 RP	19.23 (30.26)	22.5 (34.32)	0.834
SF-36 BP	29.71 (28.53)	22.5 (12.95)	0.644
SF-36 GH	38.08 (19.85)	40.75 (19.75)	0.633
SF-36 VT	56.4 (107.58)	38.38 (16.21)	0.587
SF-36 SF	53.37 (27.51)	53.75 (25.68)	0.955
SF- 36 RE	39.76 (46.22)	46.7 (48.86)	0.624
SF-36 MH	51.54 (18.71)	55.15 (21.03)	0.357
PSQI-GS	10.72 (5.26)	10.13 (3.63)	0.904

*ROCF-c* Rey-Osterrieth Complex Figure test, copy task, *ROCF-m* ROCF, memory task, *TMT-A* Trail Making Test A, attention test, *TMT-B* Trail Making Test B, divided attention test, *HADS-A* Hospital Anxiety and Depression Scale, anxiety subscale, *HADS-D* Hospital Anxiety and Depression Scale, depression subscale, *BDI-II* Beck Depression Inventory II, *SF-36 PF* 36-item Short Form Health Survey, physical functioning, *SF-36 RP* role-physical, *SF-36 BP* bodily pain, *SF-36 GH* general health, *SF-36 VT* vitality, *SF-36 SF* social functioning, *SF-36 RE* role-emotional, *SF-36 MH* mental health, *PSQI-GS* Pittsburgh Sleep Quality Index, global score

## Discussion

### Summary of results

The present study compared patients with CM to age/sex matched controls and found that patients with CM had worse cognitive and QoL profiles that improved after 3 months of preventive treatment until reaching the same scores as the controls in the cases without mood alterations.

The patients with CM included in this study scored worse than subjects in the control group on all parameters analysed for physical health (SF-36), quality of sleep (PSQI), and anxiety and depression (BDI-II). These results are consistent with those of previous studies which concluded that patients with CM have a severe functional disability that is related to their psychiatric symptoms and pain [8, 17]. Moreover, some previous studies have described how depression and anxiety are not only comorbidities of CM, they also confer greater disability in CM patients [12, 13]. This correlates with the findings of this study that showed persistent cognitive impairment despite preventive treatment in patients with a higher rate of anxiety and depression and, in addition, in patients with a lower level of education. However, when we subdivided the study sample based on anxiety and depression parameters, we found that differences in cognition between CM patients and control subjects persisted in those with a normal emotional status. This suggests that the alterations in cognition are mainly caused by the pathophysiology of CM itself and, to a lesser extent, by its comorbidities.

This study found an association between CM and poor cognitive performance at the level of attention, visual memory, and executive functions, which is consistent with some previous studies in CM. Similar findings have been reported regarding poor performance in the attention and working memory of patients with CM during the interictal period, regardless of the use of topiramate or the presence of comorbidities [16]; similarly, impairments have been reported in problem-solving and decision-making [28] and in somatosensory temporal discrimination in these patients [42].

This analysis shows that 79% of the CM patients in the sample experienced an improvement in cognitive and QoL parameters, such as bodily pain, sleep quality and general health, after 3 months of treatment with preventive therapies. Although some studies have shown that this improvement may be independent of preventive treatment as it is also observed in non-responders [43], others conducted in CM patients with comorbidities treated with onabotulinumtoxin A also described improvements in the parameters of anxiety, depression, fatigue and sleep after the use of preventive therapies [44]. Specifically, Ho et al*.*reported an improvement in cognitive status, depression, and anxiety in a sample of 60 CM patients treated with botulinum toxin, although no relationship was found between the degree of improvement at 6–12 weeks after treatment and improvement in the memory, anxiety, and depression tests analyzed [43].

This study also showed a clear improvement in all the items analysed in relation to cognition, suggesting that preventive treatment improves deficits in attention, visual memory, and executive functions, although this improvement only reached normal scores in terms of attention in the overall group of patients with CM. Subgroup analyses however showed that preventive treatment did achieve normal ranges in patients with normal mood, suggesting that, although emotional changes alone do not explain the cognitive dysfunction in CM, they may interfere with the reversibility of these alterations during preventive treatment. In this specific case, attention is the parameter that correlates most with improvement in pain and is least influenced by the other comorbidities. This is consistent with a recent study that observed that patients with migraine, and especially those with allodynia, experience a decline in attentional inhibition during the interictal phase of migraine which is independent of confounding factors such as mood disorders [45].

The oral and injectable drugs used in this study have been associated with adverse effects that may be relevant when interpreting our results. One example is the negative impact of topiramate on the concentration ability of patients [46], an adverse effect that occurs in 2% of cases and takes place during the titration period, and in a lesser manner during the maintenance period [47]. In this study, no differences were observed with respect to the preventive treatment used in relation to the reversibility of cognitive impairments or QoL, which could be due to the fact that all patients who completed the study had good tolerance to the therapies used.

The effect of time since onset of CM on cognitive performance was also analysed. We observed that patients with longer time since onset of CM obtained better scores in all the studied items than those with shorter time, although these differences did not reach statistical significance. These results coincide with previous studies in which older patients with CM showed superior cognitive performance compared to control subjects [48, 49], as well as with longitudinal studies that showed no cognitive decline over time in migraineurs [22, 23]. These findings, including those reported here, support the theory of neuronal plasticity, whereby changes in the organization of the brain’s cognitive network could constitute an adaptive factor in migraine and interfere with cortical changes that allow multimodal sensory integration, reflecting adaptation to headache recurrence [50–52].

This study has some limitations, mainly the small size of the patient sample analysed and the need to use short tests for rapid assessment in the clinic, to avoid adding a component of patient mental fatigue. In addition, there is a limitation of the learning factor in the longitudinal assessment at 3 months of treatment, which we attempted to minimize by using Taylor Complex Figure Test for longitudinal evaluation at this point. Nevertheless, even considering these limitations, the brevity of the tests makes them more applicable and generalizable to clinical practice, and in turn indicates that the changes identified are more obvious, as they are detected with screening tests without requiring an extensive neuropsychological battery. Another limitation may have been the short duration of our follow-up, which may not have been long enough to detect any other appreciable differences. It would be beneficial to increase the sample size and also the duration of the follow-up period (from 6 to 12 months) in future studies.

## Conclusions

This study confirms the cognitive impairment at the attention-perception level, divided attention, and in visual memory in patients with CM, impairments that are not explained only by the pain associated with the pathology, since they are observed in the interictal period. These impairments may show improvement in the post-treatment period following the use of preventive therapies, including better visual memory, attention, executive functions and, to a lesser extent, visual perception in patients with CM. This improvement reached the control cognition levels in patients without emotional alterations and correlates with a reduction in the impact on the QoL of migraineurs. Based on these results, modifying the initiation of a preventive treatment could reduce the interictal cognitive impairment related to CM. However, depression and anxiety could limit the reversibility of cognitive deficit. The implications of this observations are that a more routine assessment of cognitive deficits and emotional status could give a more accurate measure of cognitive performance in patients with CM and perhaps provide useful clues for finding a comprehensive treatment. Our findings confirm the need to implement effective preventive treatment early in cases of CM and to focus on their mood state.

## Supplementary Information

**Additional file 1: Supplementary Table S1.** Inclusion and exclusion criteria.

## Data Availability

The data generated during this study are available from the corresponding author upon reasonable request.
