# Supplementary material for: Preventive treatment can reverse cognitive impairment in chronic migraine
Source: J Headache Pain. 2022 Sep 15;23(1):121. doi: 10.1186/s10194-022-01486-w (PMC9476561; doi:10.1186/s10194-022-01486-w)
Supplement: Supplementary file 1 — Additional file 1: Supplementary Table S1. Inclusion and exclusion criteria. [file 10194_2022_1486_MOESM1_ESM.docx]

# Supplementary Material

## Supplementary Table S1.

Inclusion and exclusion criteria

|  | **Migraine group** | **Control group** |
| --- | --- | --- |
| **Inclusion criteria** | 1. Age 18–50 years | 1. Age 18–50 years |
|  | 1. Chronic Migraine diagnosis based on IHS criteria | 1. No migraine diagnosis |
|  | 1. Normal brain MRI scans 2. Preventive treatment initiated (treatment-naïve) or a second preventive drug was added if >50% efficacy was not achieved with previous therapies |  |
| **Exclusion criteria** | 1. Decompensated psychiatric disease or people who had experienced changes in the past 3 months if they were receiving psychiatric treatment | 1. Decompensated psychiatric disease or people who had experienced changes in the past 3 months if they were receiving psychiatric treatment |
|  | 1. Presence of oncologic, inflammatory or neurodegenerative disease | 1. Presence of oncologic, inflammatory or neurodegenerative disease |
| IHS, International Headache Society. | | |
